# Clinicopathologic features and genomic analysis of pulmonary blastomatoid carcinosarcoma

**DOI:** 10.1186/s12885-020-06748-x

**Published:** 2020-03-24

**Authors:** Jikai Zhao, Chan Xiang, Ruiying Zhao, Ping Guo, Jingjing Zheng, Han Han-Zhang, Keke Yu, Jie Zhang, Yuchen Han

**Affiliations:** 1 Department of Pathology, Shanghai Chest Hospital, Shanghai Jiao Tong University, No. 241 West Huaihai Road, Shanghai, 200030 China; 2 Burning Rock Biotech, Guangdong Province, Guangzhou, China; 3 Department of Bio-Bank, Shanghai Chest Hospital, Shanghai Jiao Tong University, Shanghai, China

**Keywords:** Blastomatoid carcinosarcoma, Pulmonary blastoma, High-grade fetal adenocarcinoma, Low-grade fetal lung adenocarcinoma, β-Catenin, *CTNNB1*

## Abstract

**Background:**

This study was designed to investigate the clinicopathologic features of pulmonary blastomatoid carcinosarcoma and explore the genomic profiles of epithelial and mesenchymal components in this tumor.

**Methods:**

Three cases of pulmonary blastomatoid carcinosarcoma were enrolled in this study. Clinicopathologic information and prognostic data were retrospectively reviewed. Diagnostic immunohistochemistry was performed. The epithelial and mesenchymal components were microdissected to investigate the genomic profiles by performing capture-based targeted next generation sequencing.

**Results:**

The epithelial components in patient one consisted of low-grade and high-grade fetal lung adenocarcinoma. Low-grade epithelial cells showed nuclear expression of β-catenin and missense mutation of *CTNNB1.* The epithelial components in another two patients consisted of high-grade fetal lung adenocarcinoma/enteric adenocarcinoma. The epithelial cells showed membrane staining of β-catenin and harbored no mutation of *CTNNB1*. The mesenchymal components in all three tumors were composed of primitive round/spindle cells without definite differentiation and showed cytoplasmic dot positive of β-catenin and no corresponding mutation. Within a tumor, both components exhibited relatively comparable molecular profile. In patient one, 4 mutations: *RB1, FAT3, PTCH1* and *LRP1B* were shared by both epithelial and mesenchymal components. Epithelial component had additional mutations in *BCOR, CTNNB1, CTCF, FAT1* and *DICER1*. In patient two, 12 mutations were shared. The epithelial component had *BRCA2* mutation and the mesenchymal had mutations in *CREBBP, ALK, DNMT3A, ASXL2, MYCN* and *RICTOR*. Patient three had 6 shared mutations. The epithelial component had an additional mutation in *KAT6A* and the mesenchymal had an additional mutation in *APC*. Collectively, we observed heterogeneity between epithelial and mesenchymal components of the same tumor.

**Conclusions:**

Blastomatoid carcinosarcoma showed characteristic morphology and immunophenotype. Parallel detection of genetic abnormalities in epithelial and mesenchymal components could provide further evidence for tumor differentiation, molecular targeting and differential diagnosis.

## Background

The concept of blastomatoid carcinosarcoma (BCS) was initially proposed by Yukio et al. in their study of classic pulmonary blastoma (CPB) and related neoplasms [1]. By definition, these tumors were categorized into one variant of carcinosarcoma with variable high-grade fetal lung adenocarcinoma (H-FLAC)/clear cell adenocarcinoma and primitive mesenchymal components. Unlike CPB, both components in BCS harbored no missense mutations of the β-catenin gene. The current WHO classification suggested these entities should be classified as carcinosarcoma and the characteristic components be mentioned in pathological report [2]. However, the exact clinicopathologic features and genomic abnormalities of BCS are still poorly understood.

In clinical practice, the diagnosis and classification of pulmonary biphasic differentiated tumors is very difficult. These entities include conventional carcinosarcoma, pulmonary blastoma, pleomorphic carcinomas and few cases of synovial sarcoma. Pulmonary blastoma (PB) can be diagnosed based on typical low-grade fetal lung adenocarcinoma (L-FLAC) and unique genetic alteration involving Wnt signaling pathway which results aberrant nuclear expression of β-catenin in both epithelial and mesenchymal cells [3, 4]. Pulmonary carcinosarcoma is defined by a mixture of non-small cell lung carcinoma and heterologous sarcomas components and can be recognized by lacking the L-FLAC and primitive mesenchymal components [5, 6]. In addition, some gene mutations, such as TP53 mutation, commonly presented in carcinosarcoma, may assist in diagnosis [7, 8]. Nevertheless, BCS seems to represent a special subtype of biphasic differentiated lung cancer, not only because of its unique morphology and immunophenotype, but also the complex molecular alterations and biological behavior. At the same time, some cases may be missed or misdiagnosed due to the disunity of diagnostic criteria.

In this study, we investigated the clinicopathologic characteristics of three cases of BCS and profiled the genetic abnormalities against epithelial and mesenchymal components. We emphasized the significance of morphologic identification and the role of immunohistochemistry and genomic analysis in auxiliary diagnosis. Besides, the treatment strategies and corresponding curative effects complemented each other, could also providing a more comprehensive understanding of BCS.

## Materials and methods

### Patients and specimens

Three cases of BCS were collected from the department of pathology of Shanghai Chest Hospital. These specimens were surgically resected between May 2012 and January 2018. All cases were processed and taken after routine internal perfusion and external fixation by 10% buffered formalin solution. Basic information of patients, grossing photographs and imaging data were reviewed from archived documents and medical records. Patients were routinely screened by chest computed tomography (CT) every six months. The median clinical follow-up time was 68 months (range 13 to 72). The last follow-up time was February 2019. Oncogenic driven gene mutation statuses, including Epidermal growth factor receptor (*EGFR*), anaplastic lymphoma kinase (*ALK*) and *ROS1* rearrangements were routinely detected and confirmed repeatedly by amplification refractory mutation system (ARMS) and/or fluorescence in situ hybridization (FISH) methods. All 3 patients had wild-type *EGFR, ALK* and *ROS1*. The pathological and clinical staging were introduced according to the recommendation of the seventh edition of lung cancer [9]. Diagnosis and recognition of H-FLAC and L-FLAC were reevaluated according to the 2015 WHO classification of lung tumors by three experienced pathologists (JZ, JKZ and YCH). Our study was approved by the ethics committee (informed consent for patient biopsy) of Shanghai Chest Hospital of Shanghai Jiao Tong University. Informed consent for surgical operation were signed by all three patients. All patients agreed to participate in the study with all relevant personal and clinical information. Written informed consent was obtained from patient 1 and verbal informed consents were obtained from immediate family members of patient 2 and patient 3 for publication of scientific papers in succession.

### Immunohistochemistry

Immunohistochemistry was performed on 4-μm dewaxing tissue slices by using the auto-stainer GI100 (DAKO OMNIS) and automated stainer (Ventana Benchmark XT; Roche Ventana) following the manufacturer’s instructions. The diagnostic primary antibodies are listed in Table 1. Normal lung tissue in specimen sections provided better negative and positive controls for panCK, TTF-1, vimentin, α-SMA and Ki-67. For each batch of samples, appropriate positive and negative controls were set according to the instructions of each antibody and the experience of our immunohistochemical laboratory. The titers of all antibodies were verified and approved by strict laboratory procedures.

**Table 1:** Immunohistochemistry antibodies used for diagnosis

Antibody	Dilution	Clone	Source	Antigen Retrieval
panCK	1:200	AE1/AE3	ChangDao	HIER
TTF-1	1:300	SPT24	LEICA	EDTA
β-catenin	1:300	β-Catenin-1	DAKO	HIER
CDX2	pre-diluted	DAK-CDX2	DAKO	HIER
CD56	1:150	123C3	DAKO	HIER
Syn	1:300	EP158	ZSGB-BIO	EDTA
INSM1	1:200	sc-271,408	SANTA CRUZ	HIER
Vimentin	1:600	V9	DAKO	HIER
α-SMA	1:300	UMAB237	ZSGB-BIO	EDTA
MyoD1	1:200	EP212	ZSGB-BIO	EDTA
S-100	pre-diluted	15E2E2 + 4C4.9	ZSGB-BIO	EDTA
Ki-67	1:300	MIB-1	ZSGB-BIO	EDTA

*CK* cytokeratin, *HIER* heat-induced epitope retrieval, *TTF-1* thyroid transcription factor-1, *EDTA* ethylenediaminetetraacetic acid, *Syn* Synaptophysin, *α-SMA* alpha smooth muscle actin

### Sequencing analysis program

#### DNA extraction and the quality assessment

For genetic analysis, hematoxylin and eosin stained sections were prepared to identify the areas of epithelial and mesenchymal components. 5~8 of 5 μm unstained tissues were then obtained using laser capture microdissection in three cases. There were very fewer L-FLAC components in patient one, therefore the total DNA of epithelial components included both low-grade and high-grade FLAC. DNA was extracted from resulting tissue fragments and paired normal lung tissue. In order to guarantee the purity of the microdissected tissues, we re-stained the remaining tumor tissue to ensure that no other ingredients doped on the target tissue. Genomic DNA was extracted with the QIAamp DNA formalin-fixed paraffin-embedded (FFPE) tissue kit (QIAGEN, Heidelberg, Germany). DNA quality was assessed by NanoDropTM 2000 (Thermo Fisher Scientific, MA, US) and agarose electrophoresis and the quantity measured by Qubit® dsDNA HS Assay Kit on Qubit® 3.0 Fluorometer (Invitrogen, CA, US).

#### NGS library preparation

DNA shearing was performed using Covaris M220, followed by end repair, phosphorylation and adaptor ligation. Fragments of size 200–400 bp were selected using Agencourt AMPure beads (Beckman Coulter, Brea, CA, USA) followed by hybridization with capture probes baits, hybrid selection with magnetic beads and PCR amplification. A bioanalyzer high-sensitivity DNA assay was performed to assess the quality and size of the fragments. 50 ng of DNA was used for library construction. Twelve PCR cycles were used for library amplification. The indexed samples were sequenced on Nextseq500 sequencer (Illumina, Inc.), San Diego, CA, USA) with pair-end reads (read length 150 bp).

#### Capture-based targeted sequencing data analysis

Sequencing data were mapped to the human genome (hg19) using Burrows-Wheeler Aligner (BWA) aligner 0.7.10 [10]. Local alignment optimization, variant calling and annotation were performed using Genome Analysis Tool Kit (GATK) v.3.2, MuTect, and VarScan [11, 12]. Variants were filtered using the VarScan fpfilter pipeline, loci with depth less than 100 filtered out. Minimal of five supporting reads were needed for INDELs and eight supporting reads were needed for SNV calling. According to the ExAC, 1000 Genomes, dbSNP, ESP6500SI-V2 database, variants with population frequency over 0.1% were grouped as SNP and excluded from further analysis. Remaining variants were annotated with ANNOVAR and SnpEff v3.6. DNA translocation analysis was performed using both Tophat2 and Factera 1.4.3 [13–15].

## Results

### Clinicopathologic features

**Fig. 1:**
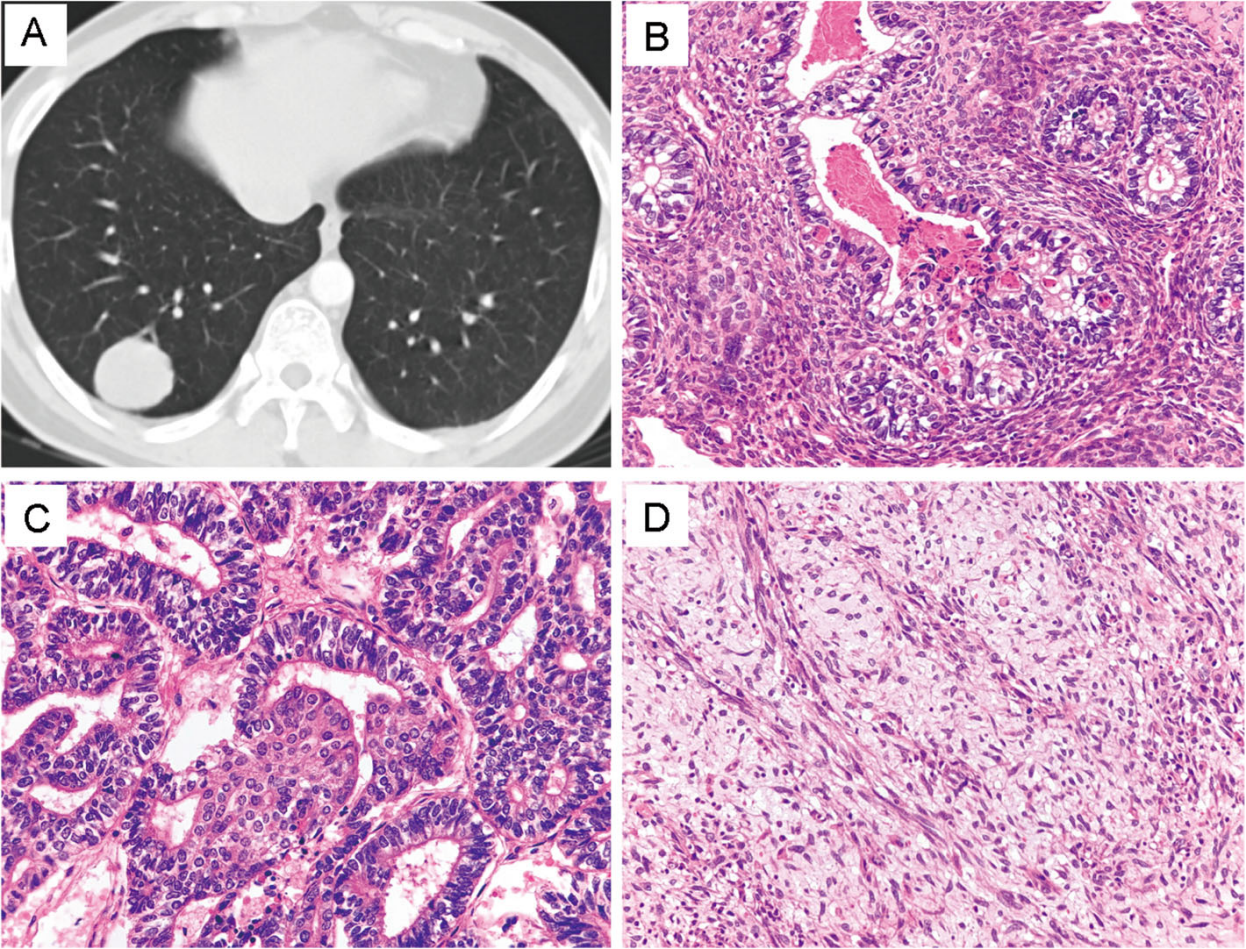
Imaging and morphology in patient one. **a** CT showed a soft tissue tumor in the right lower lobe. **b** and **c** The epithelial consisted of H-FLAC with marked pleomorphism and necrosis and mild L-FLAC with typical squamoid morules **h** and **e**, × 400). **d** Primitive mesenchymal in some areas displayed fusiform structure and mucoid stroma (H&E, × 400)

**Fig. 2:**
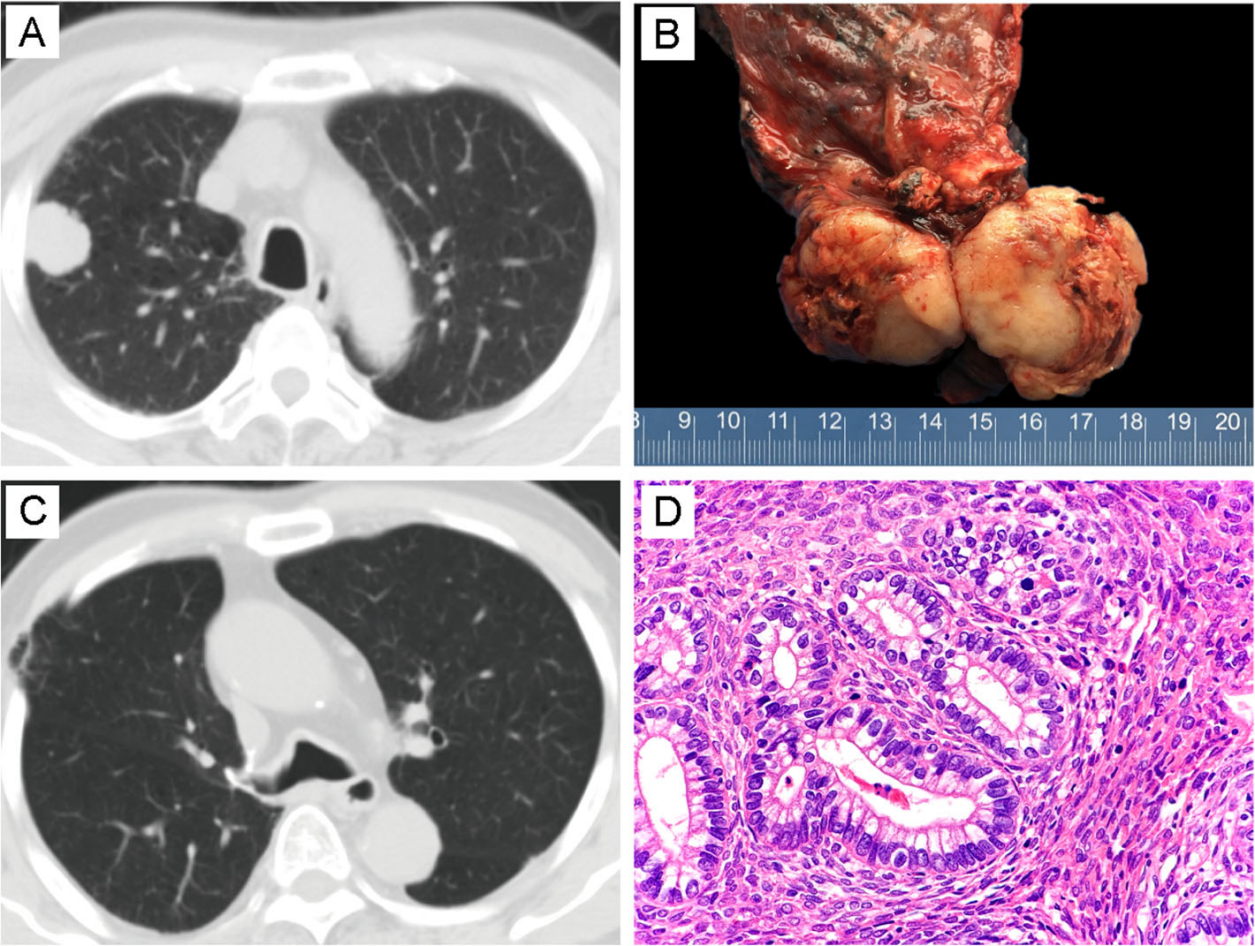
Imaging, grossing photograph and morphology in patient two. **a** CT displayed a peripheral mass in right upper lobe. **b** Gross appearance showed a well-circumscribed tumor with areas of necrosis and glistening homogeneous yellow-white cut surface. **c** CT revealed no signs of relapse in the primary site 48 months after surgical resection. **d** The tumor contained pure H-FLAC mixed with primitive stroma (H&E, × 400)

**Fig. 3:**
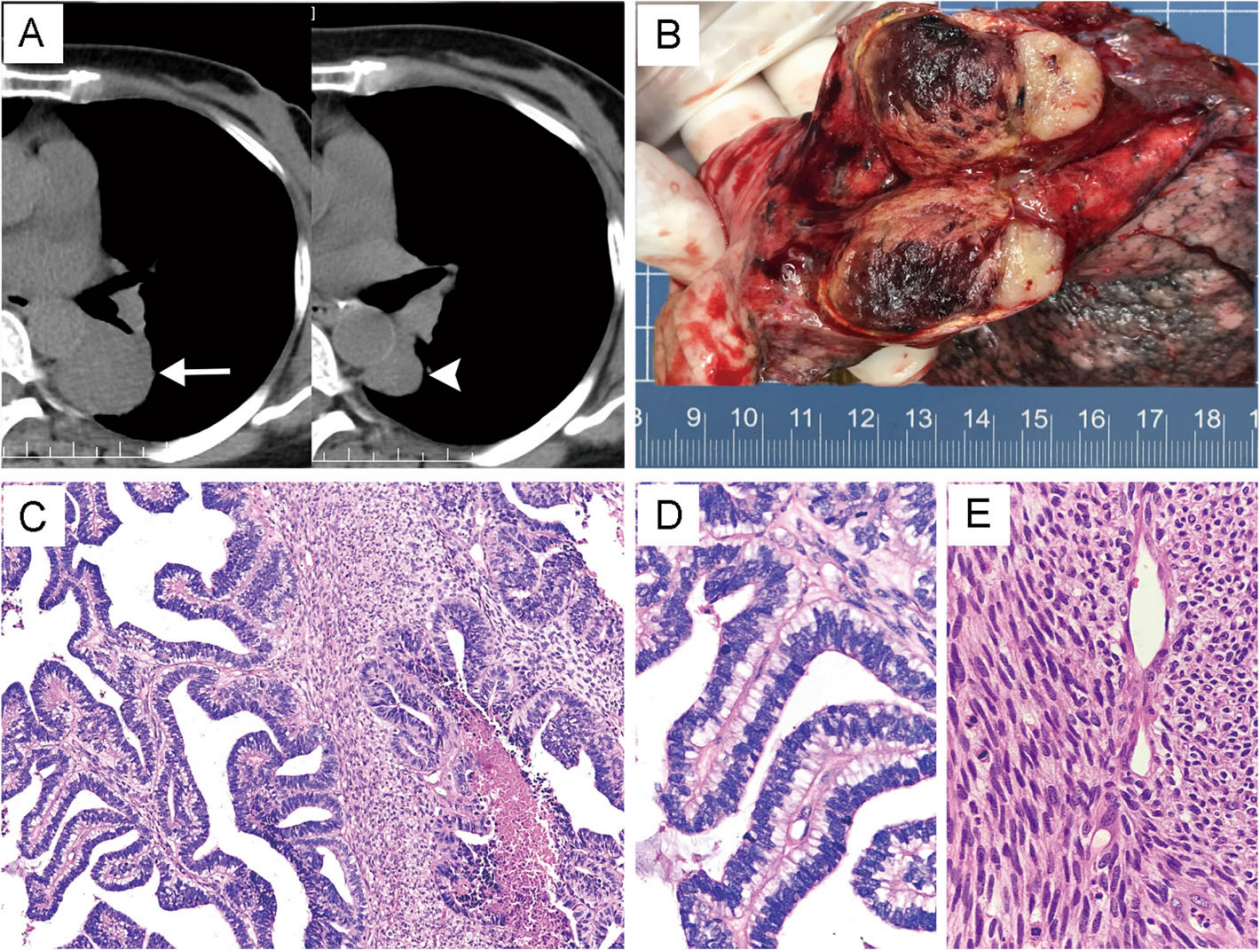
Imaging, grossing photograph and morphology in patient three. **a** Mediastinal window of CT indicated that the tumor shrank significantly before (white arrow) and after (arrowhead) chemotherapy. **b** Gross excision of the specimen showed obvious intratumoral hemorrhage. **c** The epithelial arranged in papillary structure and resembled the morphology of enteric adenocarcinoma at scanning magnification (H&E, × 100). **d-e** Higher magnification demonstrated the cytological features of epithelial and mesenchymal cells respectively (H&E, × 400)

The patients included two males and one female, with a mean age of 54 (ranged from 38 to 78). Patient one and patient two underwent lobectomy and lymph node dissection, and the first patient received three cycles of chemotherapy subsequently. The third patient underwent three courses of neoadjuvant chemotherapy followed by surgical resection. Detailed information was summarized in Table 2. CT scans revealed that all three tumors showed well-circumscribed mass. The third tumor presented a fibrous pseudocapsule and obvious hemorrhagic necrosis due to the neoadjuvant chemotherapy. Histologically, the epithelial component in patient one consisted of most H-FLAC and fewer L-FLAC components with characteristic squamoid morule structure. The mesenchymal components were mixed with epithelium, and the cells showed a short spindle or oval morphology in the myxoid background (Fig. 1). H-FLAC showed obvious nuclear atypia and mitotic activity with comedo-like necrosis. The epithelial component in patient two consisted of pure H-FLAC and the small oval stromal cells in case two were tightly arranged with high nucleocytoplasmic ratio. These cells showed no evidence of histological and immunohistochemical differentiation (Fig. 2). In patient three, the epithelial was composed of labyrinth-like glands with nucleus locating at the lateral margin and the supranuclear vacuoles toward the base. Besides, some dilated and elongated glands with dirty necrosis resembling the morphology of pulmonary enteric adenocarcinoma. The stromal elements were spindle or fibroblast-like, arranged in bundles, but structurally and morphologically insufficient to diagnose fibrosarcoma, malignant peripheral nerve sheath tumor and other special differentiated mesenchymal tumors (Fig. 3). No conventional high-grade lung adenocarcinoma or other histological non-small cell lung cancer variants were found in all three tumors. Giant cells with bizarre nucleus were not present in any of samples of mesenchymal components.

**Table 2:** Clinicopathological characteristics of three cases of pulmonary blastomatoid carcinosarcoma in this study

Case	Gender	Age ranges	Smoking status	Tumor location	Tumor size	Histology	Treatment	Stage	Follow up (status)
1	Male	35–40 (years)	no	right lower lobe peripheral	4.0 cm	mixed low-grade and high-grade fetal lung adenocarcinoma in epithelial component and primitive mesenchymal	lobectomy and lymph node dissection adjuvant three cycles of chemotherapy	T2aN0M0 IB	73 months (survival)
2	Male	75–80 (years)	50 packs/year	right upper lobe peripheral	4.2 cm	pure high-grade fetal lung adenocarcinoma and primitive mesenchymal	lobectomy and lymph node dissection	T2bN0M0 IIA	68 months (dead)
3	Female	50–55 (years)	no	left lower lobe peripheral	3.9 cm	pure high-grade fetal lung adenocarcinoma and primitive mesenchymal	chemotherapy followed by lobectomy and lymph node dissection	T2aN0M0 IB	13 months (survival)

### Immunohistochemical findings

**Fig. 4:**
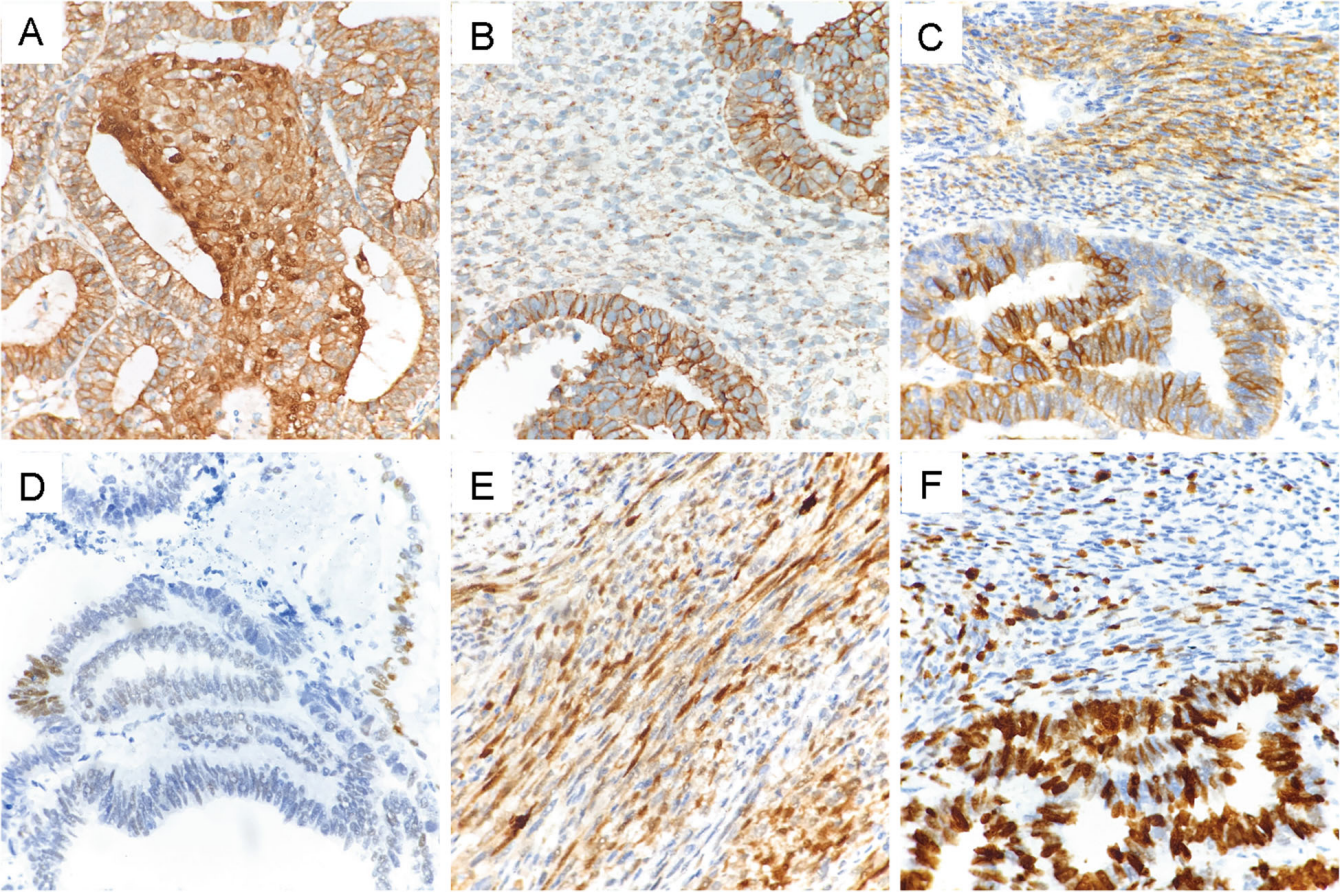
Immunohistochemical features of blastomatoid carcinosarcoma (BCS). **a** β-catenin was nucleus positive in squamoid morule cells of L-FLAC and membrane positive in columnar epithelial of H-FLAC and dot-positive in mesenchymal cells **b** (× 400). **c** Neuroendocrine marker CD56 were expressed in both epithelial and mesenchymal cells. Some epithelial cells were positive for CDX2 **d** and the mesenchymal cells was positive for S-100 **e** in the third patient (× 400). **f** The proliferation index of epithelial components was significantly higher than that of mesenchymal (× 400)

The results of immunohistochemistry were summarized in Table and Fig. 4. Only the squamoid morule cells of the L-FLAC component but not the surrounding stromal cells in patient one showed nuclear/cytoplasmic localization of β-catenin protein. All H-FLAC and mesenchymal components showed membranous/cytoplasmic dot positive. TTF-1 was positive in epithelial components of the first two tumors. Neuroendocrine markers (CD56 and Synaptophysin) showed various degrees of expression in both epithelial and mesenchymal cells, but nuclear immunostaining for INSM1 was not detected. PanCK was positive in epithelial cells and vimentin was diffusely positive in mesenchymal components. Mesenchymal markers (SMA, MyoD1) were negative in mesenchymal components. S-100 was partially expressed in mesenchymal cells in the third tumor. The proliferation index of epithelial components (30–80%) were significantly higher than that of mesenchymal cells (15–20%). The epithelial displaying enteric adenocarcinoma-like morphology was focally positive for CDX2 in the third patient.

### Genetic sequencing results

**Fig. 5:**
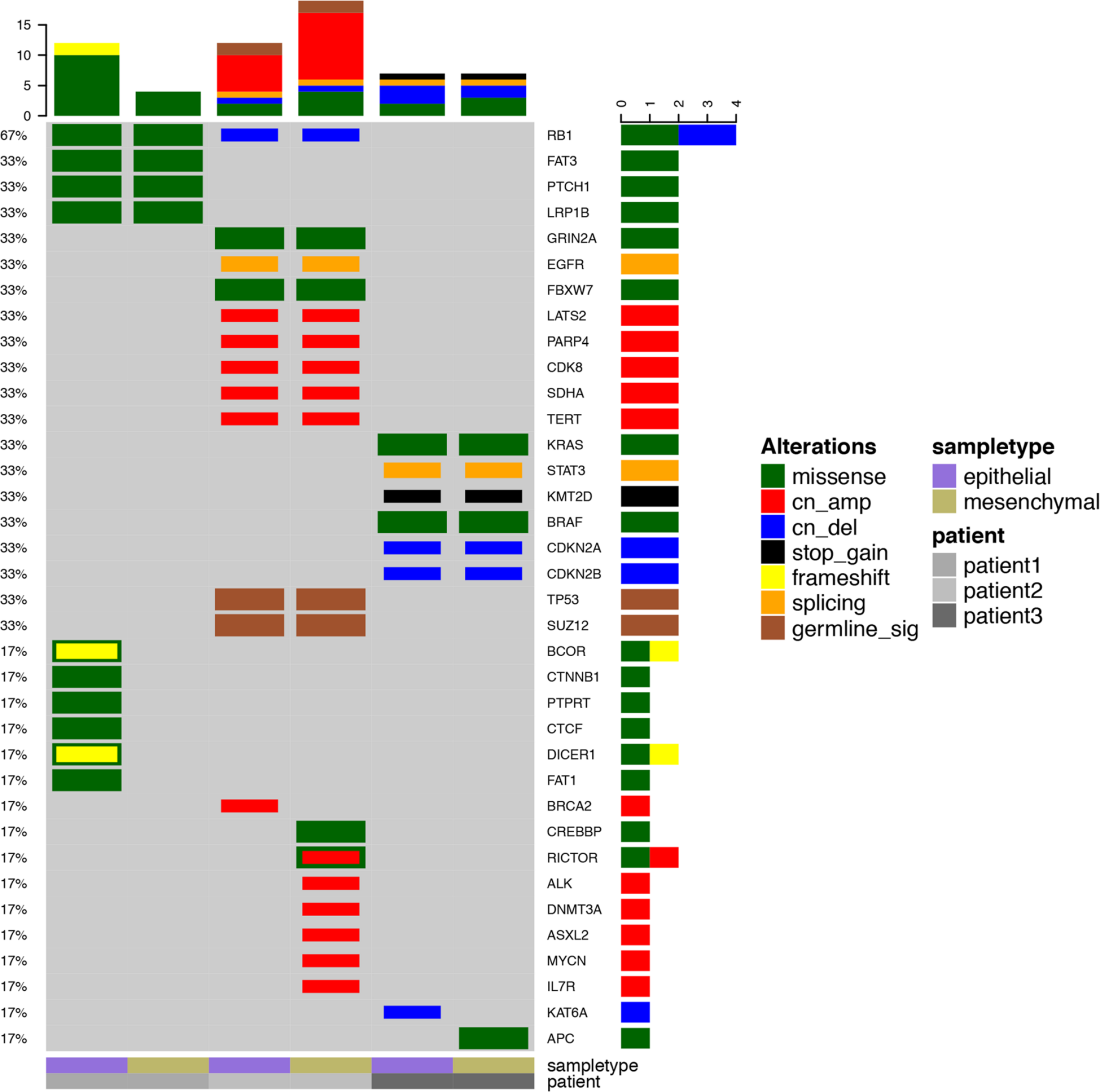
Gene mutation spectrum of blastomatoid carcinosarcoma (BCS) of three cases

Gene mutation spectrum was depicted in Fig. 5. In three tumors, epithelial and mesenchymal retained highly coincident genetic abnormalities which involved 3 groups of genes (*FAT3/LRP1B/PTCH1/RB1 in patient one, RB1/GRIN2A/FBXW7/EGFR/LATS2/PARP4/CDK8 /SDHA/TERT in patient two* and *KRAS/BRAF/STAT3/KMT2D/CDKN2A/CDKN2B in patient three*). In patient one, epithelial component harbored missense mutation of *CTNNB1* and was consistent with immunohistochemistry of aberrant nuclear localization of β-catenin in squamoid morule cells. *DICER1, BCOR, PTPRT, CTCF and FAT1* mutations only presented in epithelial component. In patient two, *BRCA2* mutation was found only in epithelial, while *ALK, RICTOR, IL7R, DNMT3A, ASXL2, MYCN* and *CREBBP* mutations were found only in mesenchymal component. In patient three, *KAT6A* mutation was found only in epithelial, while *APC* mutation was detected only in mesenchymal component, Furthermore, driver gene mutations of *KRAS* and *BRAF* were detected in patient 3. *RB1* mutations were found in the first two patients. Patient with L-FLAC component retained mostly missense mutations while patient with pure H-FLAC had various mutation types. Germline *TP53* mutation was detected in patient two and somatic *TP53* mutations were not detected in all three tumors.

## Discussion

Since Yukio et al. proposed the concept of blastomatoid carcinosarcoma (BCS) and described 5 cases of such lesions in 2004 [1], some primary biphasic differentiation lung tumors with blastomatoid mesenchymal under this terminology had been reported in succession [16–18]. Nevertheless, BCS may share similarities with pulmonary blastoma (PB) and carcinosarcoma in morphology and genetic abnormalities. This definition may cause problems in pathological diagnosis and clinical treatment.

The epithelial components of PB are essentially low-grade fetal lung adenocarcinoma (L-FLAC) and very few cases mixed with or contained pure high-grade fetal lung adenocarcinoma (H-FLAC) component [19, 20]. H-FLAC components may also present in few cases of carcinosarcoma, thus interfering with pathological diagnosis [21, 22]. Including the first defined cases, the epithelial components in reported BCS are mainly H-FLAC and/or clear cell adenocarcinoma. Other non-small cell lung cancer components, such as enteric adenocarcinoma, spindle cell and giant cell carcinoma and mucinous adenocarcinoma, have not been reported. The mesenchymal components mostly showed no tendency to maturate except for the cases reported by Sakane T et al. in which the stromal cells differentiated into chondrosarcoma [18]. And in our study, enteric adenocarcinoma was seen in one case and differentiated sarcoma components were not found.

Immunohistochemistry may be of limited use on the establishment of diagnosis, after all, there is no specific immunological markers for H-FLAC and primitive stromal cells [23]. And up to now, morphology is still the basis of pathological diagnosis for BCS. But immunohistochemical staining for Ki-67 proliferation index may be useful for distinguishing between BCS and conventional carcinosarcoma. As depicted in our cases, the epithelial and mesenchymal components respectively exhibit consistent bipolar proliferative activity. The proliferation index of epithelial components was much higher than that of mesenchymal.

Previous studies demonstrate that *CTNNB1* mutation and subsequent activation of the Wnt signaling pathway play an important role in tumorigenesis of L-FLAC and PB tumors, but not in carcinosarcoma [1, 3, 4]. Besides, few cases of PB were found to harbor somatic *DICER1* missense mutation and indicated that *DICER1* may be closely related to these tumors presenting later in life [24]. Our results demonstrated that aberrant nuclear expression of β-catenin and missense mutation of *CTNNB1* and *DICER1* were found only in L-FLAC component but not in both H-FLAC and mesenchymal components which was further supported by previous studies [25]. Moreover, our results showed that *FAT1* and *FAT3* gene missense mutations coexisted in the epithelial and mesenchymal cells in patient 1. Both *DICER1* and *FAT mutations* had not been reported in previous cases of BCS. The human *FAT1* and *FAT3* gene encode large proteins with extracellular cadherin repeats that are associated with neurodevelopment and cell migration and are most homologous, which involved in tumor suppression [26]. In addition, some genetic alterations, such as *BRCA2* and *KAT6A* only occurred in H-FLAC while *FUBP1, RICTOR,* and *CREBBP* only existed in mesenchymal components indicating the phenotypic heterogeneity among H-FLAC, L-FLAC and mesenchymal cells.

We agree that the occurrence of BCS, classical PB and carcinosarcoma are due to a group of genetic abnormalities in both epithelial and mesenchymal components [27]. Conservative or consistent genomic changes in both components play a decisive role in the development, and are also one of the criteria in molecular diagnosis for corresponding entities. At the same time, tumor cells evolve gradually with the accumulation of genetic alterations and present these intrinsic changes in morphology and immunophenotype [28].

There is still insufficient clinical evidence for standardized treatment and available recommendations for BCS and related tumors. From our clinical experience, early stage BCS tumors less than 4 cm without lymphatic and hematogenous metastasis could benefit from combined surgical resection and chemotherapy. More clinical cases, follow-up information, and comprehensive genetic analysis may be helpful for explaining the behavior of BCS and subsequent treatment strategy.

## Conclusion

Our study investigated the clinicopathologic features of pulmonary blastomatoid carcinosarcoma and compared the genetic alterations between epithelial and mesenchymal components. The results indicated that the two components retained high consistency in genetic abnormalities. we also observed heterogeneity between epithelial and mesenchymal components in the same tumor. Accurate targeting gene detection could be ancillary diagnostic techniques for BCS and provide molecular biological information for future treatment.

## Data Availability

The datasets including in this study are available from the corresponding author on reasonable request.

## Abbreviations

BCS: Blastomatoid carcinosarcoma
L-FLAC: Low-grade fetal lung adenocarcinoma;
H-FLAC: High-grade fetal lung adenocarcinoma
CPB: Classic pulmonary blastoma
ALK: Anaplastic lymphoma kinase
ARMS: Amplification refractory mutation system
FISH: Fluorescence in situ hybridization
FFPE: Formalin-fixed paraffin-embedded
CT: Computed tomography
